# Resolution of severe oncogenic hypophosphatemic osteomalacia after resection of a deeply located soft-tissue tumour

**DOI:** 10.3747/co.v16i5.412

**Published:** 2009-09

**Authors:** A.R.M. Radaideh, D. Jaradat, M.M. Abu-Kalaf, M.K. Nusier‡

**Affiliations:** * Departments of Medicine (Radaideh) and Radiology (Jaradat), Prince Basma Teaching Hospital, Ministry of Health, Irbid, Jordan; † Department of Surgery, Jordan University, School of Medicine, Amman, Jordan

**Keywords:** Oncogenic osteomalacia, mesenchymal tumour, mixed connective-tissue tumour, 1,25-dihydroxycholecalciferol, rickets, hypophosphatemia, fibroblast growth factor 23

## Abstract

Oncogenic osteomalacia is a rare metabolic bone disease characterized by phosphate leakage from the kidney and subsequent hypophosphatemia. It is caused by a phosphaturic factor produced by certain tumours. Removal of such tumours can completely cure the condition. Here, we report the case of a patient who was crippled with oncogenic osteomalacia. Extensive study revealed a tumour deeply located in the pelvis; removal of the tumour resulted in complete recovery. The tumour was identified as a mesenchymal tumour (mixed connective-tissue variant). The diagnostic evaluation, differential diagnosis, and treatment are discussed.

## 1. INTRODUCTION

Tumour-induced osteomalacia 1, also known as oncogenic osteomalacia 2, is an acquired disorder that results in markedly deranged mineral and skeletal metabolism. The disorder is characterized by hypophosphatemia (because of renal phosphate wasting), osteomalacia, bone pain, proximal muscle weakness, fractures, and functional disability 3. A reduction in circulating 1,25(OH)_2_-vitamin D despite hypophosphatemia is the biochemical hallmark of the disease 4,5. In this disorder, osteomalacia mimics the clinical phenotype of either X-linked (xlhr) or autosomal-dominant hereditary hypophosphatemic rickets (adhr) 5–8.

Oncogenic osteomalacia is commonly associated with small, slow-growing tumours of mesenchymal origin that may be difficult to detect 4,5,9. The tumours were first reported by McCance in 1947 10, and the association between those tumours and osteomalacia was reported in 1959 by Prader and coworkers 11. Normalization of serum phosphate and remission of the bone disease can be achieved if the causative tumour is located and completely removed 3,5.

Here, we report a case of oncogenic osteomalacia in a 39-year-old man with a mesenchymal tumour in the pelvis. Removal of the tumour resulted in reversal of all the biochemical abnormalities and disappearance of the clinical manifestations.

## 2. CASE REPORT

A 39-year-old man was referred from the orthopedic clinic to the endocrine clinic for re-evaluation because of suspected metabolic bone disease. His illness had started 3 years earlier, when he complained of low back pain that was progressive and associated with pain in the lower limbs and shoulders. A few months later, he developed limping on the left leg. Initially, a lumbar disease was suspected, but magnetic resonance imaging (mri) of the area was negative for disc prolapse. The patient was treated symptomatically with painkillers. Later, he complained of diffuse bone pain, accompanied by generalized muscle weakness, which led to severe debilitation. He had difficulty walking and standing from a sitting position. There was no family history of a similar condition.

Clinical physical examination revealed severe proximal muscle weakness and waddling gait. Heart, chest, and abdominal examinations were within norms, and no abnormalities were detected. Complete blood count was normal.

Biochemical evaluation revealed persistent hypophosphatemia 0.6 mmol/L (normal range: 0.81–1.6 mmol/L). Multiple determinations of serum calcium were normal: 2.1, 2.2, and 2.3 mmol/L (normal range: 2.0–2.6 mmol/L), but serum alkaline phosphatase was 839, 1910, 2093 IU/L (normal range: 100–290 IU/L). Urea at 6 mmol/L, creatinine at 60 mmol/L, and sodium and potassium levels were normal. Parathyroid hormone (pth) was 50 pg/mL (normal range: 7–53 pg/mL). Serum 25-hydroxycholecalciferol was 19 ng/L (normal range: 8.9–46.7 ng/L) and 1,25(OH)_2_- cholecalciferol was low-normal at 48 pmol/L (normal range: 48–110 pmol/L). Serum protein electrophoresis and thyroid function tests were normal. The patient’s urine was negative for protein and glucose, and no metabolic acidosis was present. Urinary inorganic phosphorus excretion was 18.5 mmol/24 h (normal: <32.3 mmol/24 h), calcium was 4 mmol/24 h (normal range: 2.5–7.5 mmol/24 h), and creatinine was 20 mmol/24 h (normal range: 7.5–17.7 mmol/24 h). The maximal rate of renal tubular reabsorption of phosphate to the glomerular filtration rate was 1.84 mg/dL (normal range: 2.3–4.3 mg/dL) 12–14.

Roentgenologic examination revealed generalized osteopenia. A three-phase ^99^Tc bone scan revealed a generalized increase in osteoblastic activity in the bony skeleton with decreased renal activity. This pattern is known as a “superscan,” which is usually seen in metabolic bone diseases, including primary and secondary hyperparathyroidism. An increase in focal activity was observed in the left calcaneus, mostly because of overuse, and in the patella of the left knee. Because of these clinical, biochemical, and Roentgenologic findings, oncogenic osteomalacia was suspected.

**FIGURE 1:**
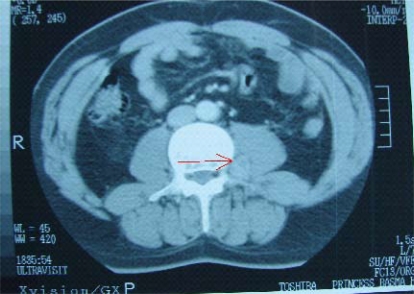
Computerized tomography image showing a well-defined (5×1.2×1-cm) enhancing soft-tissue lesion located medially of the left psoas muscle at the level of lumbar vertebra 4.

Computed tomography (ct) imaging of the neck, chest, left calcaneus, and right patella showed no soft-tissue masses to indicate the possible location of the tumour. Abdominal, renal, and pelvic ct imaging showed right middle calyceal or parenchymal calcifications. A well-defined enhancing soft-tissue-mass lesion measuring 5×1.2×1 cm was seen medially to the left psoas muscle at the level of lumbar vertebra 4 (L4). This mass contained fatty components and appeared adherent to the left transverse process of L4 (Figure 1).

**FIGURE 2:**
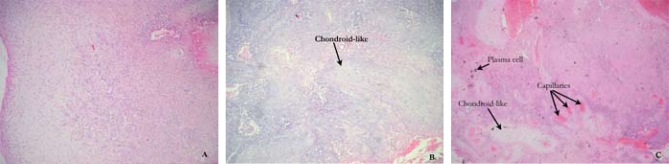
Photomicrograph of the excised mixed connective tissue (mesenchymal tumour), showing (A) plasma cells; (B) chondroid-like material; (C) plasma cells, chondroid-like material, and capillaries.

The tumour was resected on January 13, 2005, at Jordan University Hospital. Histologic examination showed an unencapsulated tumour with a variegated microscopic appearance, composed of a mixture of benign mesenchymal cells (Figure 2). Those cells were admixed with wider areas showing prominent chondroid differentiation. Islands of mature adipose tissue were also identified, as was a foci of osteoid-like matrix. The tumour contained a prominent microvasculature ranging from purely capillary-sized vessels to thicker, hyalinized ones, some of which resembled an aneurismal bone-cyst-like pattern. No evidence of malignancy was found. The pathologic diagnosis was a mesenchymal tumour (mixed connective- tissue variant), completely excised (5×5 cm) with no evidence of involvement in the excised 3 adjacent aortic lymph nodes.

Three months after surgery, the patient was asymptomatic. His weakness improved, his phosphate was normal (0.82 mmol/L), and only a slightly elevated alkaline phosphatase was recorded (300 IU/L). Eighteen months later, the patient was re-evaluated. There were no complaints, and all laboratory investigations showed values within normal ranges. Another re-evaluation, 3 years after removal of the tumour, revealed no symptoms or complaints. Laboratory investigations at that time were also normal.

## 3. DISCUSSION

The insidious development of progressive bone pain and muscle weakness is a well known feature of osteomalacia, which may have many causes 15. Diagnostic difficulties are particularly encountered in acquired hypophosphatemia, a condition associated with increased renal clearance and lower circulating 1,25(OH)_2_-cholecalciferol 4,5, especially when there is no family history of osteomalacia or rickets. Tumour-induced oncogenic osteomalacia is a rare acquired condition of metabolic bone disease, in which phosphate metabolism is markedly deranged 1,3.

To date, more than 160 cases of oncogenic osteomalacia have been reported in the literature 2,16. These tumours are usually mesenchymal or mixed connective tissue, arising from a wide range of tissues, chiefly in the head and neck, and less frequently in bones and other tissues. They are usually benign in nature. Even in histologically malignant tumours, either local recurrence or distant metastasis is extremely rare 17. The most common types are vascular neoplasms (hemangiopericytomas). Other types include fibromas, chondrosarcomas, histiocytomas, neuroblastomas, and prostate carcinoma 2,4,5,9,18.

The clinical signs and symptoms of oncogenic osteomalacia as a neoplastic syndrome are nonspecific and often lead to erroneous diagnosis of joint, muscle, or neurologic disorders. Most patients report bone or muscle tenderness and weakness. Our patient complained of diffuse progressive bone pain over 3 years and had difficulty walking.

The biochemical markers of oncogenic osteomalacia include hypophosphatemia, hyperphosphaturia, decreased tubular phosphate reabsorption, increased serum alkaline phosphatase in the presence of normal calcium, 25(OH) vitamin D, and normal or slightly elevated serum pth 5. Selective low and low-to-normal 1,25(OH)^2^ vitamin D and hypophosphatemia are biochemical features also seen in adhr and xlhr 5, in which they present a lifelong clinical and biochemical picture. The management of these two conditions is different, with the clinical picture in the current case resolving after removal of the phosphaturic mesenchymal tumour, mixed connective-tissue variant 2, which may indicate involvement of the phosphate-regulating pathway 5.

In the current case, as in others previously described, not all biochemical markers were present 5. The late presentation and absence of family history, together with cure after surgery, made a diagnosis of adhr or xlhr unlikely in our patient. There was no role for adjuvant therapy. Treatment with large doses of neutral phosphate presents great risk for the development of tertiary hyperparathyroidism in oncogenic osteomalacia 4. Detection of the tumour responsible therefore held great clinical importance. Because these tumours are usually small in size, they are difficult to identify. In our patient, the tumour was found after almost total body ct imaging and was confirmed by mri. Similar diagnostic procedures, and other newer techniques such as combined positron-emission tomography–ct, are used to detect these tumours 19. In our patient, the tumour was located deep in the pelvis in a very unusual location—a location that, to our knowledge, has not previously been described 2. The tumour was a phosphaturic mesenchymal tumour, mixed connective-tissue variant type, as are 80% of such tumours 2. The complete resolution of the clinical and biochemical abnormalities after removal of the tumour indicates that it was the source of the hormonal factor that inhibited Na–P co-transport in renal proximal tubular cells and lowered 1,25(OH)_2_ vitamin D production 1,4.

Patients with oncogenic osteomalacia have been found to have high serum levels of fibroblast growth factor 23 (fgf23). Evaluation of this substance and of the phosphate-regulating gene with homologies to endopeptidase on the X chromosome (*PHEX*), whose loss of function causes adhr and whose mutation causes xlhr, may provide diagnostic clues (Table I) 4,5,20. Unfortunately, fgf23 and *PHEX* were not evaluated because of an impossibility of performing immunohistochemistry at the time.

## 4. CONCLUSIONS

For the first time in Jordan, we describe a patient with oncogenic osteomalacia, stemming from an unusually deeply located mesenchymal tumour in the pelvis. Complete reversal of the clinical and biochemical manifestations was achieved after removal of the tumour. For patients who have a clinical and biochemical picture suggestive of oncogenic osteomalacia, it is of crucial importance to perform a meticulous survey to detect the tumour or tumours responsible.

## Figures and Tables

**Table I:** Hypophosphatemic conditions caused by excess action of fibroblast growth factor 23 (fgf23) and causes of excess fgf23 action

Disease	Causes of excess fgf23 action
Autosomal dominant hypophosphatemic rickets or osteomalacia	Dysregulated fgf23 expression because of mutations in the fgf23 gene
	Overexpression of fgf23 in bone because of mutations in the dmp1 gene
X-linked hypophosphatemic rickets or osteomalacia	Overexpression of fgf23 in bone because of mutations in pehx gene
McCune Albright syndrome and fibrous dysplasia	Overexpression of fgf23 in bone
Tumour-induced rickets or osteomalacia	Overexpression of fgf23 in the responsible tumour

*dmp1* = dentin matrix acidic phosphoprotein 1; *phex* = phosphate-regulating endopeptidase homolog, X chromosome–linked.
